# Background noise inhibits listeners' use of contextual cues for dysarthric speech

**DOI:** 10.1121/10.0042316

**Published:** 2026-02-02

**Authors:** Katerina A. Tetzloff, Sarah E. Yoho, Eric W. Healy, Stephanie A. Borrie

**Affiliations:** 1 Department of Linguistics, Stony Brook University, Stony Brook, New York 11794, USA; 2 Department of Speech and Hearing Science, The Ohio State University, Columbus, Ohio 43210, USA; 3 Department of Speech and Hearing Sciences, Utah State University, Logan, Utah 84322-1000, USA

## Abstract

Contextual clues aid in speech perception, especially when the signal is degraded by speech disorders or background noise. This study examined whether different types of degradation affect how listeners use contextual predictability. Two groups of 50 listeners were tested across three conditions: dysarthric speech, neurotypical speech masked by noise, and dysarthric speech masked by noise. Listeners relied on semantic context similarly for dysarthric speech in quiet and neurotypical speech in noise (single degradations). However, when dysarthric speech was masked by noise (concurrent degradation), contextual benefit was greatly reduced. Findings highlight the communication burden noise adds for understanding dysarthric speech.

## 1. Introduction

Contextual information—semantic cues surrounding a word or phrase—helps listeners infer meaning, particularly when speech is degraded. Such predictability supports speech perception for signals disrupted by external factors like background noise (Hutchinson, 1989; Nittrouer and Boothroyd, 1990; Dubno *et al.*, 2000; Bradlow and Alexander, 2007) or inherent factors like vocoding (Sheldon *et al.*, 2008; Kong *et al.*, 2015; Patro and Mendel, 2016) and pathological speech disorders such as dysarthric speech (Garcia and Cannito, 1996; Carter *et al.*, 1996). However, it is unclear whether different types of degradation differentially affect the benefit listeners gain from contextual predictability.

Research has shown that sentence-level predictability aids perception of dysarthric speech. For severely impaired speakers, acoustic distortion can be so extreme that context provides little benefit (Yorkston *et al.*, 1991). Conversely, when impairment is mild, acoustic cues may be sufficient, reducing reliance on context (Carter *et al.*, 1996). Similar patterns are observed with other degraded signals; contextual benefit is greatest at moderate levels of distortion, where neither the signal nor context is entirely dominant (e.g., Golestani *et al.*, 2009; Sohoglu *et al.*, 2012; Bhandari *et al.*, 2021).

Dysarthric speech differs from other degraded signals because it disrupts both segmental and suprasegmental cues. These complex, variable disruptions to articulation, rhythm, intonation, and voice quality reduce acoustic predictability, resulting in increased ambiguity to the meaning of words. One way in which listeners perceive ambiguous or degraded speech is through learning the distributional regularities of speech signals over time, mapping the limited acoustic input of degraded speech to stored mental representations of intact speech (Diehl *et al.*, 2004; Dahan and Magnuson, 2006; Clayards *et al.*, 2008; Bybee, 2010; Cutler, 2012; Wiener *et al.*, 2019). The disruptions of dysarthric speech may reduce the ability to achieve these mappings of degraded input-to-mental representations and thus limit listeners' ability to use statistical knowledge of speech cues to take advantage of contextual information within an utterance.

In contrast, neurotypical speech masked by noise retains its internal structure and is therefore highly redundant in terms of predictable segmental and suprasegmental cues. Most real-world noises are spectrotemporally complex, meaning that they vary in both time and frequency, and listeners can exploit brief spectrotemporal glimpses—portions of speech momentarily unmasked—to access reliable cues (Miller and Licklider, 1950; Buss *et al.*, 2004; Cooke, 2006; Fogerty *et al.*, 2018). This indicates that the statistical regularities of speech that has retained its internal structure provide cues when listening in complex noise, as the portions of the target signal that are spared from masking match existing and expected mental representations of speech.

This study examined whether pathological degradations in dysarthria limit listeners' use of contextual information compared to external degradation from noise. Previous work has shown no effect of the type of modulated noise on use of contextual information during speech perception (Carter *et al.*, 2022), so cafeteria noise was chosen for the present study, as it represents a typical spectrotemporally complex noise environment that listeners may encounter in their day-to-day lives. Two experiments were conducted with sentences varying in semantic predictability, spoken by a neurotypical speaker and a speaker with moderate dysarthria. In experiment 1, dysarthric speech was presented in quiet and neurotypical speech in noise. In experiment 2, both speech types were presented in noise. Comparing these conditions allowed us to assess contextual use under inherent degradation (dysarthria), external degradation (noise), and combined degradation (dysarthria in noise). We hypothesized that dysarthria, especially when combined with noise, would reduce listeners' reliance on semantic context because of greater mismatch between the signal and their expectations (Van Engen and Peelle, 2014).

## 2. Method

### 2.1 Speech Stimuli

The speech stimuli consisted of 100 sentences from the Revised Speech Perception in Noise (SPIN; Bilger *et al*., 1984), corpus, which is a widely-used and well validated set of sentences designed specifically to examine the role of semantic context in speech perception under challenging listening conditions. The corpus uses final-word scoring, which allows the semantic predictability of the scored word to be carefully controlled in each sentence. Half of these SPIN sentences were produced by a 63-year-old female speaker with moderate spastic dysarthria secondary to a cerebral vascular accident (i.e., stroke). Her speech was characterized perceptually by slow speaking rate, equal and excess stress, imprecise articulation, and a strained–strangled vocal quality. The dysarthria diagnosis and severity estimate was made by three independent speech-language pathologists with expertise in differential diagnosis of motor speech disorders. The other half of these SPIN sentences were produced by a 66-year-old female control (neurotypical) speaker having no history of neurological or speech impairment. For each speaker, half (*n* = 25) of the sentences were from the &high predictability' subsets of SPIN corpus (e.g., “*The dog gave a warning growl*.”), and half were “low predictability” (e.g., “*We could discuss the dust*.”), in which the final word used for scoring each sentence differed in the degree to which it could be predicted by prior sentence context. Both speakers had standard American English dialects. Each phrase of the 48 kHz, 16-bit speech stimuli was equated to within ±1 dB based on total root mean square amplitude (i.e., amplitude normalization). For the noise conditions, a cafeteria noise from  Auditec (2015) was used. This real-world recording was made in a busy hospital cafeteria and overdubbed three times to increase sound density. For experiment 1, the neurotypical speech was presented at a signal-to-noise ratio (SNR) of 0 dB, and the dysarthric speech was presented in quiet. For experiment 2, the neurotypical speech was again presented at a SNR of 0 dB, and the dysarthric speech was presented at a SNR of +5 dB. These SNRs were selected to ensure that scores in all conditions (those for both speech types and both contexts) remained free of floor and ceiling effects and in the linear portion of the psychometric function relating SNR to intelligibility. A common SNR capable of meeting this requirement likely does not exist because any single SNR would likely produce either ceiling effects for the neurotypical speech or floor effects for the dysarthric speech; we note that the SNRs used (0 and +5 dB) are among the most commonly encountered in noisy everyday environments (Pearsons *et al.*, 1977; Smeds *et al.*, 2015; Mansour *et al.*, 2021).

### 2.2 Procedures

Prior to beginning the study, all participants gave their informed consent (Utah State University Institutional Review Board, Protocol No. 14439). The experiment was hosted on the Gorilla (2020) platform, which is a web program specifically designed to collect behavioral data from online participants, and administered through Prolific (2023), which is an online crowdsourcing website that hosts experiments and manages a pool of suitable research participants for various types of studies.

Before initiating the listening portion of the experiment, all participants completed a demographic questionnaire that involved questions pertaining to their age, sex, race, ethnicity, history of hearing loss, and history of speech-language disorders. The participants then received instructions on how to conduct the listening task, including the required use of headphones; they were able to set the volume themselves. They were then told that they would hear a series of sentences spoken by two individuals, an individual with no speech disorder but with background noise present and an individual with a speech disorder (with or without background noise, depending on the experiment). They were instructed to type only the final word that they heard in each sentence and to guess to the best of their ability if they were unsure, or to put an X if they were unable to guess. All stimuli were presented in a different randomized order for each participant, and all participants heard all 100 sentences (25 high predictability and 25 low predictability from both the dysarthric and neurotypical speaker). The total duration of the experiment was approximately 30 min, and no participant was common across the two experiments.

### 2.3 Participants

Fifty listeners participated in experiment 1. All reported living in the United States and being monolingual native speakers of American English; participants were filtered on Prolific (2023) for these criteria. All participants reported no known history of hearing loss or speech-language disorder. Their mean age was 37.2 years [standard deviation (SD) = 12.3], and 23 (46%) were female. Data for eight participants were excluded because of poor compliance, operationally defined as no response to 20% or more of the stimuli (*n* = 8). Thus, the final data set for experiment 1 is based on 42 participants.^1^

An additional group of 50 listeners participated in experiment 2. Like the listeners in experiment 1, all reported living in the United States and being monolingual speakers of American English, as filtered by Prolific (2023). No participants reported a known history of hearing loss or speech-language disorder. Their mean age was 43.8 years (SD = 13.8) Thirty-four (68%) were female, 15 (30%) were male, and one (2%) preferred not to say. Eleven people were excluded for poor compliance (*n* = 39).

## 3. Results

Intelligibility data were derived from the orthographic transcriptions that were scored as either correct or incorrect using Autoscore (Borrie *et al.*, 2019), which is an open-source tool used for automatically scoring orthographic transcripts. Words were considered correct if they matched the final-word target exactly, or if they differed only in plurality or tense. A percent words correct (PWC) score was then calculated for each listener participant for each of the listening conditions.

### 3.1 Experiment 1

**Fig. 1.**
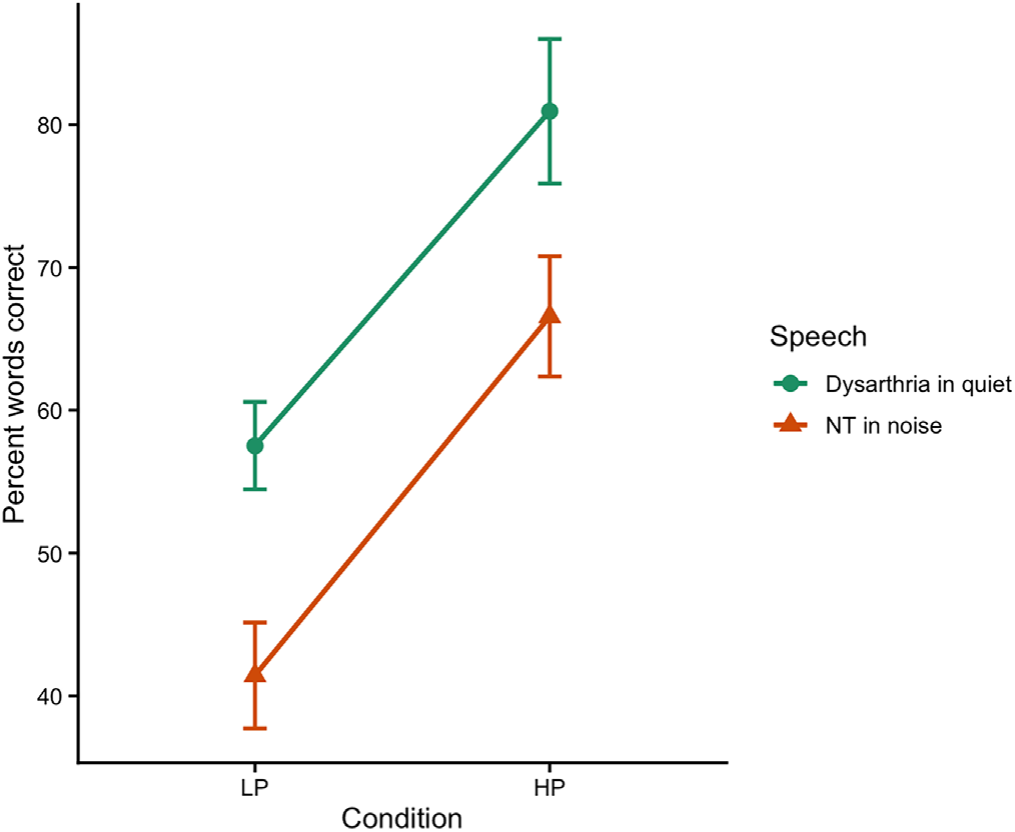
LP, low predictability; HP, high predictability; NT, neurotypical. Interaction plot showing the effect of sentence predictability (LP versus HP) on word recognition accuracy across speech types (dysarthria in quiet versus NT speech in noise). Error bars represent ±1 standard error of the mean.

Figure 1 displays group mean intelligibility scores (in PWC) for each of the conditions of experiment 1. The figure shows that scores were higher for dysarthric speech (69% overall) than for the neurotypical speech at the SNR used (54% overall) but that all scores are free of floor and ceiling effects. However, more importantly, the difference in scores across high versus low predictability sentences (indicative of the use of semantic context) was similar for both dysarthria (23% points) and for speech in noise (26% points).

All statistical analyses were run in r using packages lme4 (Bates *et al.*, 2015) and emmeans (Lenth, 2025). For experiment 1, we first fit a mixed-effects linear model including speech type (dysarthria in quiet, neurotypical in noise), predictability (low, high), and their interaction with a random by-participant intercept. Main effects were assessed from the interaction model using estimated marginal means.

Neurotypical speech in noise was significantly less intelligible than the dysarthric speech in quiet for both high predictability sentences (ß = –14.38, *p* < 0.0001) and low predictability sentences (ß = –16.10, p < 0.0001). Additionally, across speech types, low predictability sentences were significantly less intelligible than high predictability sentences (ßs > 23.42, *p*s <  0.0001). However, more importantly, the interaction between speech type and predictability and was non-significant (ß = 1.71, *p* = 0.54), indicating that listeners used contextual information to a similar degree when perceiving both dysarthria and neurotypical speech in noise.

To further examine the use of contextual information across speech types, the correlation between the context difference scores for individual listeners (the difference between high and low predictability in PWC) for dysarthria and the context difference scores for speech in noise was calculated. There was a moderate positive correlation between the difference scores across speech type (*r* = 0.46, *p* = 0.002), indicating that listeners who were better able to take advantage of contextual information when perceiving one type of degraded speech were also better able to take advantage of contextual information when perceiving the other type of degraded speech.

### 3.2 Experiment 2

**Fig. 2.**
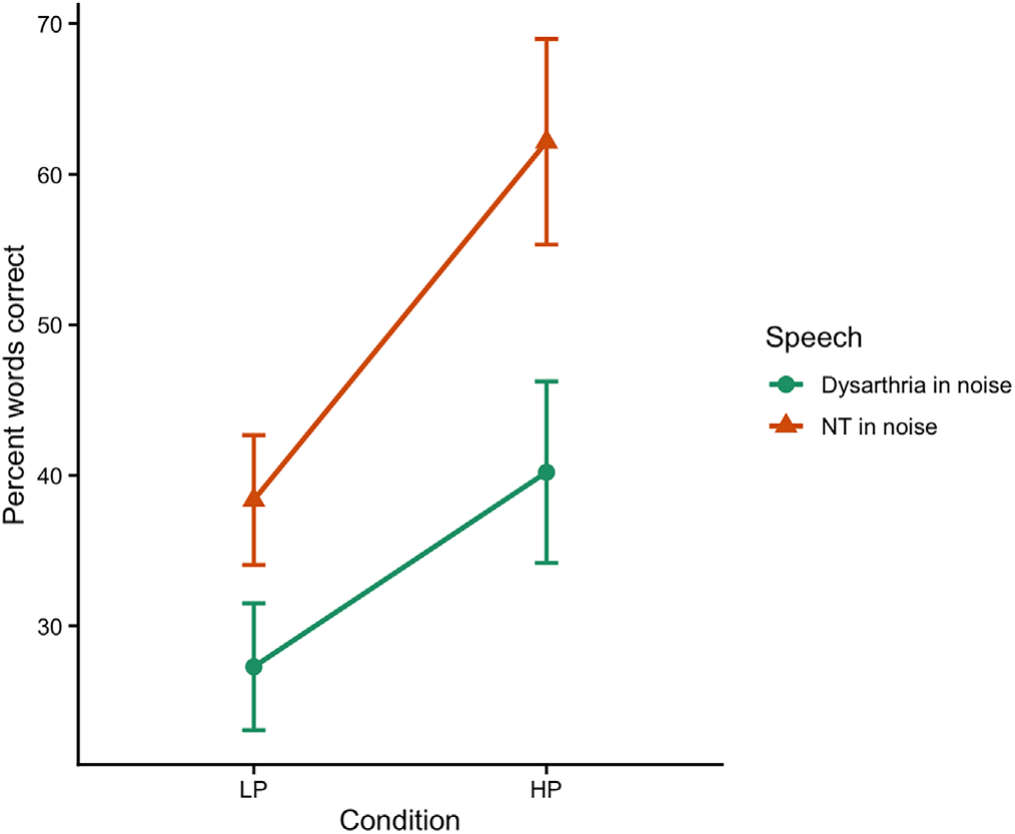
LP, low predictability; HP, high predictability; NT, neurotypical. Interaction plot showing the effect of sentence predictability (LP versus HP) on word recognition accuracy across speech types (dysarthria in noise versus NT speech in noise). Error bars represent ±1 standard error of the mean.

Figure 2 again displays group mean intelligibility in each condition. In these conditions, dysarthria in noise (34% overall) was below scores for typical speech in noise (50% overall), but again, all scores are free of floor and ceiling effects. More importantly, the drop from high predictability to low predictability scores (use of context) was found to be similar to that observed in experiment 1 for typical speech in noise (26% points in experiment 1 and 24% points here in experiment 2). However, for dysarthria in noise, this use of context measure was considerably smaller (13% points).

A second linear mixed-effects model was run, parallel to that above. In experiment 2, neurotypical speech in noise was significantly more intelligible than the dysarthric speech in noise for low predictability (ß = 11.08, *p* < 0.0001) and high predictability (ß = 21.95, *p* < 0.0001) sentences. Again, high predictability sentences were more intelligible than low predictability sentences across both types of speech (ß > 12.92, *p* < 0.0001). However, more importantly, there was a significant interaction between speech type and predictability (ß = 10.87, *p* = 0.0006) (Fig. 2), such that the difference between low and high predictability sentences was greater for neurotypical speech in noise versus dysarthric speech in noise.

The correlation for experiment 2 again examined the relationship between the context difference scores for individual listeners (the difference between high and low predictability in PCW) for dysarthria in noise and the context difference scores for neurotypical speech in noise. There was a moderate positive correlation between the difference scores across speech type (*r* = 0.36, *p* = 0.03), which suggests that listeners who were better able to use contextual information with one type of degraded speech were also better to use this information to accurately perceive the other type of degraded speech.

## 4. Discussion

This study examined how listeners use contextual information to understand speech degraded by two different mechanisms, *inherent degradation* from moderate spastic dysarthria and *external degradation* from background noise. Experiment 1 showed that listeners used context similarly for both dysarthric speech in quiet and neurotypical speech in noise, despite the significant perceptual differences and nature of the disruptions across the two types of speech degradation. The dysarthric speech was characterized by slow rate, imprecise articulation, and poor vocal quality, whereas the neurotypical speech in noise required listeners to “glimpse” unmasked portions of the signal (Cooke, 2006; Apoux and Healy, 2013; Fogerty *et al.*, 2018). It might be expected that listeners would rely more on context for neurotypical speech in noise because of its greater acoustic predictability and their everyday experience with this type of degradation. In contrast, dysarthria introduces unpredictability and unfamiliar distortions to both prosodic changes and articulatory cues, potentially making the surrounding context less reliable because of a mismatch between the signal and listeners' expectations. Surprisingly, the results showed equivalent contextual benefit, possibly suggesting a global role of contextual predictability that supports perception across different degraded speech types.

In experiment 2, adding background noise to both speech signals altered this pattern. When dysarthric speech was masked by noise, listeners' ability to benefit from context dropped sharply. This indicates that background noise disproportionately impairs contextual processing for pathologically degraded speech. These findings align with prior research showing that contextual benefit declines with increasing signal disruption (Golestani *et al.*, 2009; Obleser and Kotz, 2011; Bhandari *et al.*, 2021) and with work demonstrating that even mild dysarthria is highly vulnerable to noise (Yoho and Borrie, 2018; Chiu and Neel, 2020; Borrie *et al.*, 2023). Importantly, this highlights real-world challenges for individuals with dysarthria and their communication partners, as noisy environments not only decrease intelligibility but also reduce listeners' ability to use semantic cues to support understanding.

Across both experiments, individual differences emerged; listeners who were strong users of context were consistent across conditions, regardless of degradation type. This finding parallels evidence linking cognitive abilities such as working memory (Lee *et al.*, 2014) and vocabulary knowledge (McLaughlin *et al.*, 2018) to speech perception under challenging conditions. Vocabulary knowledge, in particular, has been linked to perception of dysarthric speech (McAuliffe *et al.*, 2013), foreign-accented speech (Banks *et al.*, 2015), and speech in noise (Carroll *et al.*, 2016). Although not directly measured here, higher-level language and cognitive skills likely contribute both to overall speech perception and to effective use of context in degraded listening situations, which warrants further investigation.

These findings are also consistent with prior work showing that listeners who are good at perceiving dysarthric speech tend to be the same listeners who are good at perceiving speech in noise (Borrie *et al.*, 2017), and this relationship remains even when the listeners have significant sensorineural hearing loss (Yoho *et al.*, 2023). This suggests a shared underlying ability to decode degraded speech signals, independent of the source of disruption.

The study's primary limitation is the use of a single talker for each speech type. Although common in speech perception research, results may vary across dysarthria subtypes (e.g., hypokinetic, ataxic) or severity levels, as different speech characteristics may interact with noise in distinct ways. Future work should explore a range of dysarthria severities and noise conditions. Additionally, because dysarthric speech is highly susceptible to masking, future studies should examine how hearing loss impacts listeners' ability to leverage context. Listeners with sensorineural hearing loss are particularly susceptible to the presence of background noise (e.g., Festen and Plomp, 1990; Bacon *et al.*, 1998, Kenyon *et al.*, 1998), and understanding the combined effects of disordered speech, environmental noise, and listener hearing status could inform more effective clinical strategies. Because communication is a two-way process, research that integrates both talker and listener factors will be essential to improving real-world outcomes.

## 5. Conclusions

Normal hearing listeners relied on context equally for dysarthric speech and neurotypical speech in noise. However, when noise was added to dysarthric speech, contextual benefit was significantly reduced. Moreover, individuals' ability to use context was consistent across speech types, suggesting a general listener skill in leveraging semantic context cues. These findings underscore the profound challenges faced by people with dysarthria and their partners in noisy environments, where both intelligibility and contextual support are compromised.

## Data Availability

The data and code for this study are openly available at https://osf.io/96ctz/.

## Footnotes

^1^
Recent work by Ziegler *et al.* (2021) has shown that nine crowdsourced participants are sufficient for this type of online speech perception task.
